# Prevalence of Precancerous Conditions and Gastric Cancer Based upon the National Cancer Screening Program in Korea for 7 Years, Single Center Experience

**DOI:** 10.1155/2015/571965

**Published:** 2015-01-06

**Authors:** Ji Hyuk Kang, Yun Jeong Lim, Jung Hyun Kang, Jae Nam Yang, Seung Min Shin, Jae Hyeuk Choi, Jin Ho Lee

**Affiliations:** Department of Internal Medicine, Dongguk University Ilsan Hospital, Dongguk University College of Medicine, 27 Dongguk-ro, Ilsandong-gu, Goyang-si, Gyeonggi-do 410-773, Republic of Korea

## Abstract

*Aims*. Gastric cancer is the second most prevalent cancer and the third leading cause of cancer-related deaths in Korea. The National Cancer Screening Program (NCSP) has implemented esophagogastroduodenoscopy (EGD) biennially for all Koreans starting in their 40s. This study was conducted to estimate the clinical relevance of NCSP through identifying the prevalence of gastric disease, including cancer. *Materials and Methods*. Data from 40,821 subjects who received the screening EGD in the single center for 7 years were retrospectively investigated. *Results*. The overall prevalence of nonatrophic/atrophic/metaplastic gastritis, peptic ulcer, adenoma, early gastric cancer (EGC), and advanced gastric cancer (AGC) was 44.28%, 27.97%, 14.95%, 0.59%, 0.43%, 0.21%, and 0.09%, respectively. The prevalence of metaplastic gastritis, peptic ulcer, adenoma, EGC, and AGC was significantly higher in men than in women. The prevalence of preneoplastic/neoplastic disease significantly increased with age. Judged from the ratio of EGC to AGC, the proportion of EGC made up to 70% of all cancers. *Conclusions*. Screening endoscopy starting for people in their 40s should be strongly recommended for the elderly. Through the NCSP, the early detection of gastric cancer might contribute to the decreased mortality rate due to gastric cancer in Korea.

## 1. Introduction

Gastric cancer is the second most common cancer in Korea [1]. With the introduction of the National Cancer Screening Program (NCSP), all Koreans receive an esophagogastroduodenoscopy (EGD) or an upper gastrointestinal series biennially, starting at 40 years old. The idea behind the NCSP was that early detection of gastric cancer at a curable stage could eventually reduce mortality rates in Korea due to the disease. Early gastric cancer (EGC) can be completely cured with endoscopic submucosal dissection (ESD) and laparoscopic gastrectomy [2–4]. The NCSP was prompted in 1999 partly due to the ratio of EGC to advanced gastric cancer (AGC) of 14.9%–28.6% in 1995 and 32.8% in 1999, reflecting the gradual rise in the incidence of EGC and the decreased incidence of AGC [5]. Progression of gastric cancer is influenced by environmental factors, such as* Helicobacter pylori* (*H. pylori*) infection, smoking, alcohol consumption, salt intake, and consumption of smoked or burnt food, all of which affect the progression of atrophic gastritis, intestinal metaplasia, dysplasia, and adenocarcinoma [6, 7].

The present study analyzed EGD reports on NCSP examinees in the single center over 7 years. The aim of this study was to investigate the distribution of gastric diseases (atrophic gastritis, intestinal metaplasia, dysplasia, and gastric cancer) and the prevalence of EGC and AGC.

## 2. Materials and Methods

The electronic medical records of 40,821 patients who had undergone EGD at the single university hospital from October 2005 to April 2012 were retrospectively analyzed. Subjects previously diagnosed with stomach cancer or an adenoma had been excluded. Gastric diseases were categorized as nonatrophic gastritis, atrophic gastritis, metaplastic gastritis, adenoma, EGC, AGC, peptic ulcer, and others. Atrophic gastritis involves loss of glandular cells due to chronic inflammation of gastric mucosa. Endoscopically, the submucosal blood vessels are readily apparent because the mucous membrane becomes thinner. Metaplastic gastritis involves the transformation of gastric epithelial cells to intestinal cells due to chronic inflammation and is evident as bumpy nodules in EGD. EGC was diagnosed when the adenocarcinoma was localized in the gastric mucosa or the submucosa layer, regardless of lymph node metastasis, and AGC was categorized if the cancer cells penetrated the proper muscle layer. The institutional review board of our institution reviewed and approved this study.

## 3. Statistical Analysis

The Chi-square test was used for the verifications. The statistical significance was set at *P* < 0.05. SPSS version 12.0 for Windows (SPSS, Chicago, IL, USA) was used for analysis.

## 4. Results

**Figure 1:**
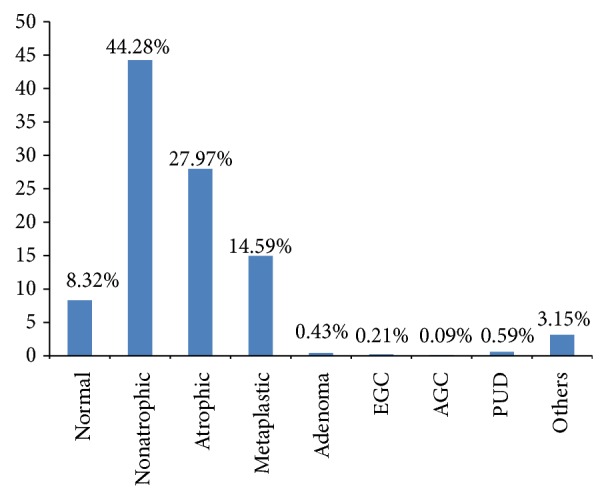
Overall prevalence of gastric diseases. EGC: early gastric cancer; AGC: advanced gastric cancer; PUD: peptic ulcer disease.

Of the 40,821 examinees, 17,807 (43.62%) were men and 23,014 (56.38%) were women. The subjects ranged in age from 40 years to 93 years, with a mean age of 56.23 ± 9.76 years (Table 1). Regarding the prevalence of gastric diseases, 18,076 people (44.28%) had nonatrophic gastritis, 11,419 (27.97%) had atrophic gastritis, 6,102 (14.95%) had metaplastic gastritis, 175 (0.43%) had adenoma, 86 (0.21%) had EGC, 37 (0.09%) had AGC, and 241 (0.59%) had peptic ulcers. Among peptic ulcers, 102 people (0.25%) had gastric ulcers, 96 (0.24%) had duodenal ulcers, and 43 (0.1%) had both gastric ulcers and duodenal ulcers (Figure 1). Women made up the majority of nonatrophic and atrophic gastritis cases, while more men had metaplastic gastritis, adenomas, EGC, and AGC. Endoscopy showed that normal gastric mucosa decreased as people aged, while nonatrophic gastritis did not remarkably change in age groups. The prevalence of atrophic and metaplastic gastritis increased in people 70 years and over but decreased in people over 80 years. Adenoma and gastric cancer increased in older subjects, with highest prevalence in subjects in their 80s.

**Figure 2:**
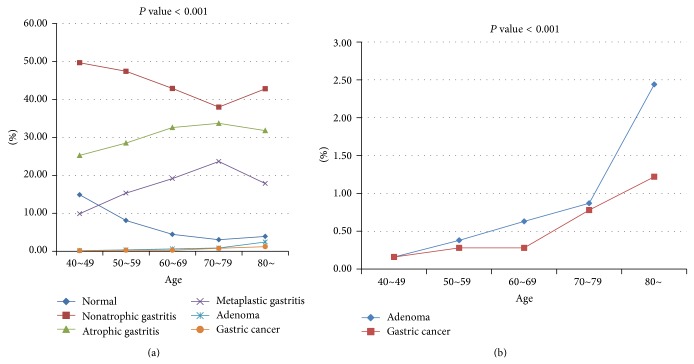
(a) Prevalence of gastric disease according to age distribution. (b) Prevalence of adenoma and gastric cancer according to age distribution.

**Figure 3:**
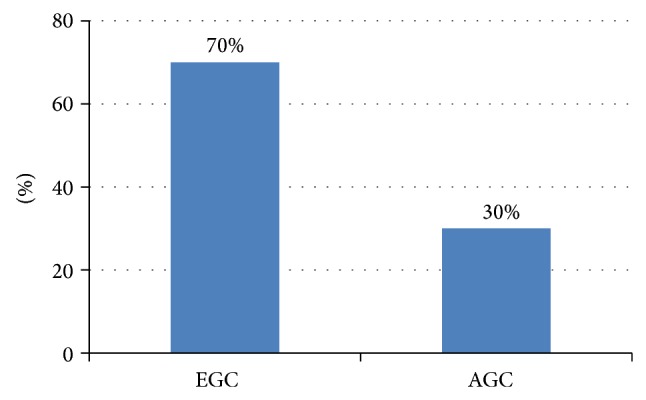
Proportion of early gastric cancer and advanced gastric cancer among gastric cancer diagnosed through National Cancer Screening Program from October 2005 to April 2012. EGC: early gastric cancer; AGC: advanced gastric cancer.

In particular, the prevalence rate of atrophic gastritis and metaplastic gastritis was highest in individuals in their 70s. In comparison, the highest prevalence of adenomas and gastric cancer was in subjects over 80 years (Figure 2). One hundred twenty-three gastric cancers (0.3%) were diagnosed through NCSP during the 7-year study period. The incidence rate of EGC and AGC was 0.21% (*n* = 86) and 0.09% (*n* = 37), respectively. EGC comprised 70% of the gastric cancers (Figure 3), and the EGC/AGC ratio was 2.32 (86/37).

## 5. Discussion

Gastric cancer is the second most common cancer in Korea [1]. Screening for the early detection of gastric cancer can reduce gastric cancer-related morbidity and mortality and increases the likelihood of curing gastric cancer by noninvasive methods, including ESD and laparoscopic surgery. In Korea, the NCSP was introduced in 1999 to detect the five most common cancers and implemented biennial EGD for men and women older than 40 years. The 10-year survival rate from EGC exceeds 90%, while the 5-year survival rate of AGC was less than 50~60% [2–4]. To reduce gastric cancer-related mortality, early detection of adenoma or EGC is important. National screening for gastric cancer is also done in Japan, and since its introduction, the incidence and mortality of gastric cancer have decreased [8].

Considering the high frequency of intestinal metaplasia in gastric cancer patients, atrophic gastritis and metaplastic gastritis are understood as prodromal signs of gastric cancer [9]. Atrophic gastritis leads to loss of the glandular cells and is diagnosed pathologically. However, in the present study, nonatrophic gastritis, atrophic gastritis, and metaplastic gastritis were endoscopically diagnosed, leading to interobserver differences. Since metaplastic gastritis shows prominent nodularity, such a bias is likely relatively negligible compared to nonatrophic/atrophic gastritis. Human gastric carcinogenesis follows the sequences of atrophic gastritis that destroys the glandular functions, metaplasia, dysplasia, and, finally, adenocarcinoma [6, 7]. Considering that gastric carcinogenesis takes place, EGD observation of metaplastic gastritis may help the patient and doctor stay alert to the increased risk for gastric cancer [9]. Also, endoscopic treatment of adenoma can prevent gastric cancer. In the present study, the prevalence of atrophic and metaplastic gastritis was 42.92%, which is similar to other reports [10–13]. In this study, metaplastic gastritis, adenomas, and gastric cancer were significantly higher in men than in women. This may reflect that men are more likely to be exposed to risk factors, including* H. pylori* infection, drinking, and smoking [14, 15].

Elderly was defined as more than 65 years of age and very elderly as more than 85 years of age. EGD is safe and effective for elderly patients [16, 17]. There was no information in the literature on the mortality in the elderly and very elderly in association with EGD, but tachyarrhythmia, transient bradycardia, brief confusion, and bronchospasm occurred more frequently in the elderly and very elderly [18, 19]. In the present study, the prevalence of atrophic gastritis and metaplastic gastritis increased with advanced age (70–79 years), and the prevalence of adenoma and gastric cancer increased with advanced age (over 80 years).

**Figure 4:**
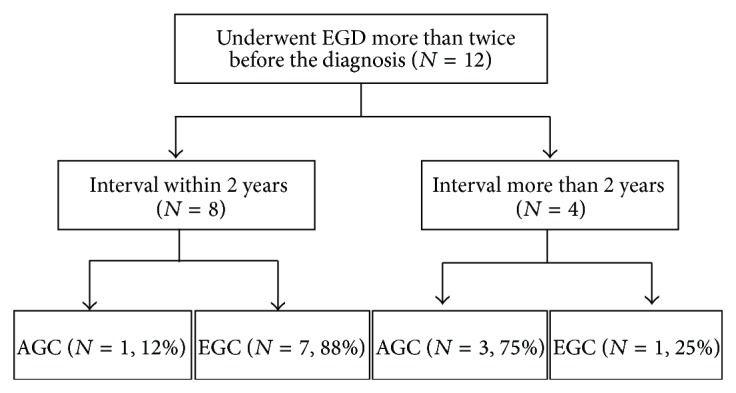
Proportion of early gastric cancer and advanced gastric cancer according to screening interval for gastric cancer. EGD: esophagogastroduodenoscopy; EGC: early gastric cancer; AGC: advanced gastric cancer.

A 10-year study of the natural progression of EGC reported that 64.28% (36/56) of the patients progressed to AGC [20, 21]. Determining the proper screening interval is important. All the gastric cancer patients who underwent EGD every 2 years were confirmed to have had EGC and displayed a 5-year survival rate of 96.5%, which was significantly greater than the 71.0% in the EGD group that underwent EGD less frequently [22]. No significant difference in the EGC/AGC ratio was observed between subjects receiving EGD within 2-year interval than every 2 years and every 2 or more years (88.9% versus 83.3%), but the ratios that could be endoscopically treated were 29.6% (8/27) and 0% (0/12), respectively (*P* = 0.0344) [23]. EGC was observed in 96% (25/26) of the patients who underwent screening EGD within 2 years, whereas only 71% (34/48) of the patients who undergo the screening EGD at more than 2 years were confirmed to have EGC (*P* = 0.01) [24]. In the present study, of the subjects who were diagnosed with gastric cancer, 12 patients underwent EGD more than twice before the diagnosis. Of those 12, 8 underwent EGD within 2 years: one was diagnosed with AGC and seven were diagnosed with EGC. Of the other 4 subjects who underwent EGD less than once every 2 years before they were diagnosed as gastric cancer, one was diagnosed with EGC and three were diagnosed with AGC (Figure 4). Gastric cancer patients who underwent EGD less than once every 2 years have favorable prognosis. In high-risk examinees diagnosed with a precancerous lesion in their EGD or who had symptoms or a family history of gastric cancer, it may be necessary to have an EGD which may be necessary more than once every 2 years.

In conclusion, a screening EGD should be actively performed for subjects over 40 years old and especially for the elderly (over 65 years old) because advanced age increases the risk of the gastric cancer. Through the NCSP, early detection of gastric cancer likely contributes to the decreasing mortality rate due to gastric cancer in Korea.

## Figures and Tables

**Table 1:** Demographic characteristics of the study population.

Age (years)	Males, *n* (%)		Females, *n* (%)		Total, *n* (%)	
Mean age ± SD	57.19 ± 10.03		55.49 ± 9.47		56.23 ± 9.76	
40–49	4,544	(11.1)	6,935	(17.0)	11,479	(28.1)
50–59	6,618	(16.2)	8,931	(22.0)	15,549	(38.2)
60–69	4,131	(10.1)	4,959	(12.2)	9090	(22.3)
70–79	2,254	(5.5)	2,012	(4.9)	4,266	(10.4)
80-	260	(0.6)	177	(0.4)	437	(1.0)

Total	17,807	(43.5)	23,014	(56.5)	40,821	(100)

Values are presented as mean ± SD or *n* (%).
